# METTL3 relieved the injury of SH‐SY5Y cells treated with lipopolysaccharide and exposed to sevoflurane through regulating the m6A levels of Sox2

**DOI:** 10.1002/brb3.2936

**Published:** 2023-03-28

**Authors:** Yilong Wang, Zeyong Yang

**Affiliations:** ^1^ Department of Anesthesiology, International Peace Maternity and Child Health Hospital Shanghai Jiao Tong University School of Medicine Shanghai China; ^2^ Shanghai Key Laboratory of Embryo Original Diseases Shanghai China; ^3^ Shanghai Municipal Key Clinical Specialty Shanghai China

**Keywords:** METTL3, postoperative cognitive dysfunction, sevoflurane, Sox2

## Abstract

**Introduction:**

Postoperative cognitive dysfunction (POCD) is a common complication of the central nervous system in elderly patients. The objective of this study was to investigate the role of methyltransferase 3 (METTL3) in the POCD progression.

**Methods:**

The SH‐SY5Y cells were treated with lipopolysaccharide (LPS) and exposed to sevoflurane to establish a POCD cell model. The cell viability and proliferation were assessed with MTT and EdU assays. Besides, the cell apoptosis was determined with TUNEL staining and flow cytometry. Additionally, the inflammatory factors were measured with ELISA. N6‐methyladenosine (m6A) RNA Methylation Quantification Kit was used to detect the m6A levels. The relative expressions of methyltransferase 3 (METTL3) and Sex‐determining region Y‐box‐2 (Sox2) was measured with RT‐qPCR and western blot assays. RNA methylation immunoprecipitation‐real‐time quantitative PCR was performed to detect the RNA that was m6A modified.

**Results:**

After LPS treatment and sevoflurane exposure, the cell viability and proliferation were decreased and the cell apoptosis was elevated. The m6A and the METTL3 expression levels in the POCD cell model were declined. METTL3 overexpression promoted the cell growth and inhibited the cell apoptosis in the POCD cell model. Besides, the Sox2 levels were reduced in the POCD cell model. METTL3 silencing declined the m6A and mRNA levels of Sox2, while overexpression of METTL3 elevated it. The relationship between METTL3 and Sox2 was confirmed with double luciferase assay. Finally, Sox2 silencing neutralized the role of METTTL3 overexpression in the POCD cell model.

**Conclusion:**

METTL3 relieved the injury of the SH‐SY5Y cells induced by LPS treatment and sevoflurane exposure through regulating the m6A and mRNA levels of Sox2.

## Graphical abstract

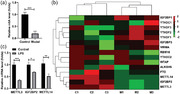

## 1 INTRODUCTION

Postoperative cognitive dysfunction (POCD) is a common complication of the central nervous system in elderly patients (Evered & Silbert, 2018). POCD is often manifested in mental disorder, anxiety, personality change, memory, abstract thinking, and orientation, accompanied by the decline of social activity (Lin et al., 2020). Studies have shown that POCD or delirium occurs in approximately 40% of the 16 million elderly Americans undergoing surgery every year, which is related to the decline of life quality, mortality, and the increased risk of Alzheimer's disease (Berger et al., 2019). The incidence of POCD in China is 15%, while the incidence of POCD in elderly patients over 60 years old is higher than 25% 1 week after operation (Chi et al., 2017; Wang et al., 2021). POCD of elderly patients will prolong the hospitalization and rehabilitation time, increase the medical expenses, and bring a heavy burden to the society. Therefore, effective strategies for the prevention of POCD in elderly patients is of positive significance.

N6‐methyladenosine (m6A), a methylation at the N6 position of adenosine, has been considered as the most abundant epigenetic modification in eukaryotic mRNA since its discovery in the 1970s (Feng et al., 2018). m6A modification affects nearly every stage of mRNA metabolism, including pre‐mRNA splicing, mRNA transport from the nucleus to the nucleus, mRNA translation efficiency, mRNA stability, and the subcellular localization (Zhao et al., 2017). Methyltransferase 3 (METTL3) is the core component of the m6A methyltransferase complex, responsible for catalyzing the formation of mRNA m6A in eukaryotes (Li et al., 2019). Accumulating researches demonstrated METTL3 plays a crucial role in the pathogenesis of neurodegenerative diseases, such as Alzheimer's disease (Han et al., 2020) and Parkinson's disease (Qin et al., 2020). He and Wang (2021) found that the perturbation in m6A methylation levels and decrease of METTL3 was occurred in the POCD mice. However, the precise mechanism of METTL3 in POCD progression remains unclear.

Sex‐determining region Y‐box‐2 (Sox2) is an important member of Sox gene family, which is located on human chromosome 3q26.33–3q27. The expression product of Sox2 is a protein containing 317 amino acid residues, with a relative molecular weight of about 37,000 da (Li et al., 2020). Early studies showed that Sox‐2 has a dynamic expression pattern during embryonic development and organogenesis. It can be used as a marker of embryonic pluripotency and maintain the multidirectional potential of embryonic stem cells (Akiyama et al., 2016; Jeon et al., 2020). As reported by Gui et al. (2021), Sox2 was demonstrated to be closely relative to the learning and memory in POCD progression.

Thus, we constructed a POCD cell model by lipopolysaccharide (LPS) and sevoflurane stimulation. This study aimed to explore the specific role of METTL3 in POCD and the interaction relationship between METTL3 and Sox2.

## 2 MATERIAL AND METHOD

### 2.1 Cell culture and POCD model establishment

The human neuroblastoma SH‐SY5Y cells were provided by Procell Life Science & Technology Co., Ltd. (Wuhan, China). SH‐SY5Y cells were seeded in DMEM/F12 medium containing 10% fetal bovine serum and 1% penicillin and streptomycin, and cultured in a 37°C, 5% CO_2_ incubator. The POCD model establishment was performed according to a previous study (Jeong et al., 2019). Briefly, the cultured cells were treated with 1 μg/mL LPS and exposed to 4% sevoflurane for 1 h.

### 2.2 Cell transfection and RNA sequencing

The cultured cells were transfected with METTL3 overexpressed vector (OE‐METTL3) and empty vector (OE‐NC); small interfering RNA METTL3 (si‐METTL3) and small interfering RNA negative control (si‐NC); and small interfering RNA Sox2 (Si‐Sox2) and Si‐NC. All these plasmids were provided by GENEWIZ (Suzhou, China) and transfected into the cells using the Lipofectamine 2000 kit following the protocols. Additionally, the differentially expressed genes in the OE‐METTL3‐transfected SH‐SY5Y cells were analyzed using RNA sequencing technology (Keyan Biology, Nanjing, China).

### 2.3 MTT assay

After cell counting, SH‐SY5Y cells with a density of 2 × 10^5^ cells/well were placed in 96‐well plates and cultured in a 5% CO_2_, 37°C incubator for 6 h. Then, 100 μL medium containing 10% MTT was added to each well of the plate, and the cells were cultured for another 4 h. Finally, 100 μL of dimethyl sulfoxide reagent was added to each well, and the absorbance was measured with a microplate reader (BIORAD, USA) at the wavelength of 492 nm. The OD value was recorded to calculate the cell viability.

### 2.4 EdU assay

The SH‐SY5Y cells were seeded on cell slides in 24‐well plates at a density of 1 × 10^5^ cells/well, and the plates were incubated at 37°C for 24 h. After that, 10 μmol/L EdU was added to each well of the plate for 24 h. Cells were fixed in 40 g/L paraformaldehyde for 15 min and permeabilized with 0.5% Triton X‐100 for 20 min. Nuclei were stained with Hoechst33342. After completing all steps, the positive cells with fluorescent signal were observed by a fluorescence microscope (Leaica, Germany).

### 2.5 Cell apoptosis determination

The SH‐SY5Y cells in each group were collected and resuspended to the density of 2.5 × 10^5^/mL. A total of 200 μL of cell suspension was inoculated into six‐well plates and cultured for 72 h. After washing with binding buffer, another 250 μL binding buffer was added to resuspend cells. Then, 5 μL Annexin V‐FITC and 10 μL PI (20 μg/mL) were added, and the cells were incubated at room temperature for 10 min in the dark. Thereafter, the apoptosis rate was detected by a BD‐Verse flow cytometry (BD, USA).

### 2.6 TUNEL staining

The cells of each group were centrifuged to discard the culture medium, and the cells of each treatment group were fixed with 4% formaldehyde at 20°C. After fixation, cells were washed with phosphate‐buffered saline three times. Afterward, according to the instructions of the TUNEL‐DAPI double staining kit, cells were first blocked with 3% H_2_O_2_ for 15 min. After washing with phosphate‐buffered saline, the 0.1% TritonX‐100 was added and incubated for 8 min at 4°C. After washing again, the cells were added with TUNEL reaction solution in the dark at 37°C for 1 h. Then the cells were stained with 0.5 μg/mL 4′,6‐diamidino‐2‐phenylindole dihydrochloride for 5 min. A fluorescence microscopy was used to detect the apoptosis of cells.

### 2.7 RT‐qPCR

According to the instructions of the RNA extraction and cDNA synthesis kit (Vazyme, Nanjing, China), the RNA of SH‐SY5Y cells was extracted and reverse transcribed into cDNA. The SYBR Green Mix kit (Vazyme) was used to perform the qPCR reaction in an ABI7500 FAST system. Reaction conditions was set as follows: 25°C for 2 min; 95°C predenaturation for 2 min; denatured at 95°C for 30 s; and annealed at 60°C and extended for 30 s, a total of 40 cycles. The 2–ΔΔCT method was used to calculate the relative expression of mRNA and GAPDH was used as the internal control.

### 2.8 Western blot

SH‐SY5Y cells were lysed with RIPA buffer (Beyotime) to isolate the total proteins. After the determination of protein concentration with a BCA kit (Beyotime), the proteins were separated using 10% SDS‐PAGE, followed by transferring to PVDF membrane (Beyotime). After that, the membranes were incubated with primary antibodies (anti‐Bax, anti‐Bcl‐2, anti‐caspase3, anti‐Sox2, anti‐METTL3, and anti‐GAPDH; Abcam, USA) at 4°C overnight. Subsequently, the membranes were incubated with HRP‐labeled secondary antibody (Abcam) for 1 h. Finally, the membranes were visualized using immobilon ECL kits (Beyotime). Image J software was used to calculate the gray value of each band.

### 2.9 Luciferase reporter assay

According to the prediction results, we synthetized the wild‐type (WT) and mutant (MUT) fragment (500–1600 bp) of Sox2 and inserted into luciferase reporter vector. The WT and MUT fragment of Sox2 was synthetized by General Bio (Anhui) and inserted into Pmir‐GLO vectors. The SH‐SY5Y cells were co‐transfected with WT or MUT vectors along with OE‐METTL3 or Sh‐METTL3 using Lipofectamine 3000 for 48 h. The luciferase activity was detected using the dual‐luciferase reporter assay system.

### 2.10 RNA methylation immunoprecipitation‐real‐time quantitative PCR assay

RNA methylation immunoprecipitation‐real‐time quantitative PCR experiments were performed according to the instructions of the MagnaRIP™ RNA‐Binding Protein Immunoprecipitation Kit (Millipore, USA). A total of 1 × 10^6^ SH‐SY5Y cells were inoculated into a 10‐cm‐diameter cell culture dish, and then lysed with 700 μL RIP lysis buffer on ice for 10 min and centrifuged at 14,000 × *g* for 10 min. Next, the supernatant was collected and washed with wash buffer. The cell lysis was incubated with magnetic beads at room temperature for 1 h and divided into IgG group (added with Normal Rabbit IgG antibody) and m6A group (added with anti‐m6A antibody). Then, the magnetic bead/antibody mixture was incubated with the cell lysate overnight at 4°C. The next day, magnetic beads was harvested and treated with proteinase K. The RNA in the complex pulled down was extracted as mentioned above, and reverse transcribed into cDNA. The Sox2 levels were detected using RT‐qPCR assay.

### 2.11 M6A methylation level determination

The M6A methylation levels were determined with EpiQuik a M6A RNA Methylation Quantification Kit (AmyJet Scientific Inc, Wuhan, China). All operations were carried out according to the protocols of the kits.

### 2.12 Inflammatory factor detection

The TNF‐α, IL‐1β, and IL‐6 levels in the SH‐SY5Y cells were assessed with the corresponding ELISA kits (Beyotime, Shanghai, China) in accordance with the instructions of the kits.

### 2.13 Statistical analysis

SPSS 22.0 software was used for data analysis. The data were expressed by mean ± SD. The student *t*‐test was used for the comparison between the two groups, and the one‐way ANOVA was used for the comparison between multiple groups. A *p*‐value of <.05 was considered statistically significant.

## 3 RESULTS

### 3.1 LPS stimulation inhibited the cell growth and induced the cell apoptosis of the SH‐SY5Y cells

**FIGURE 1:**
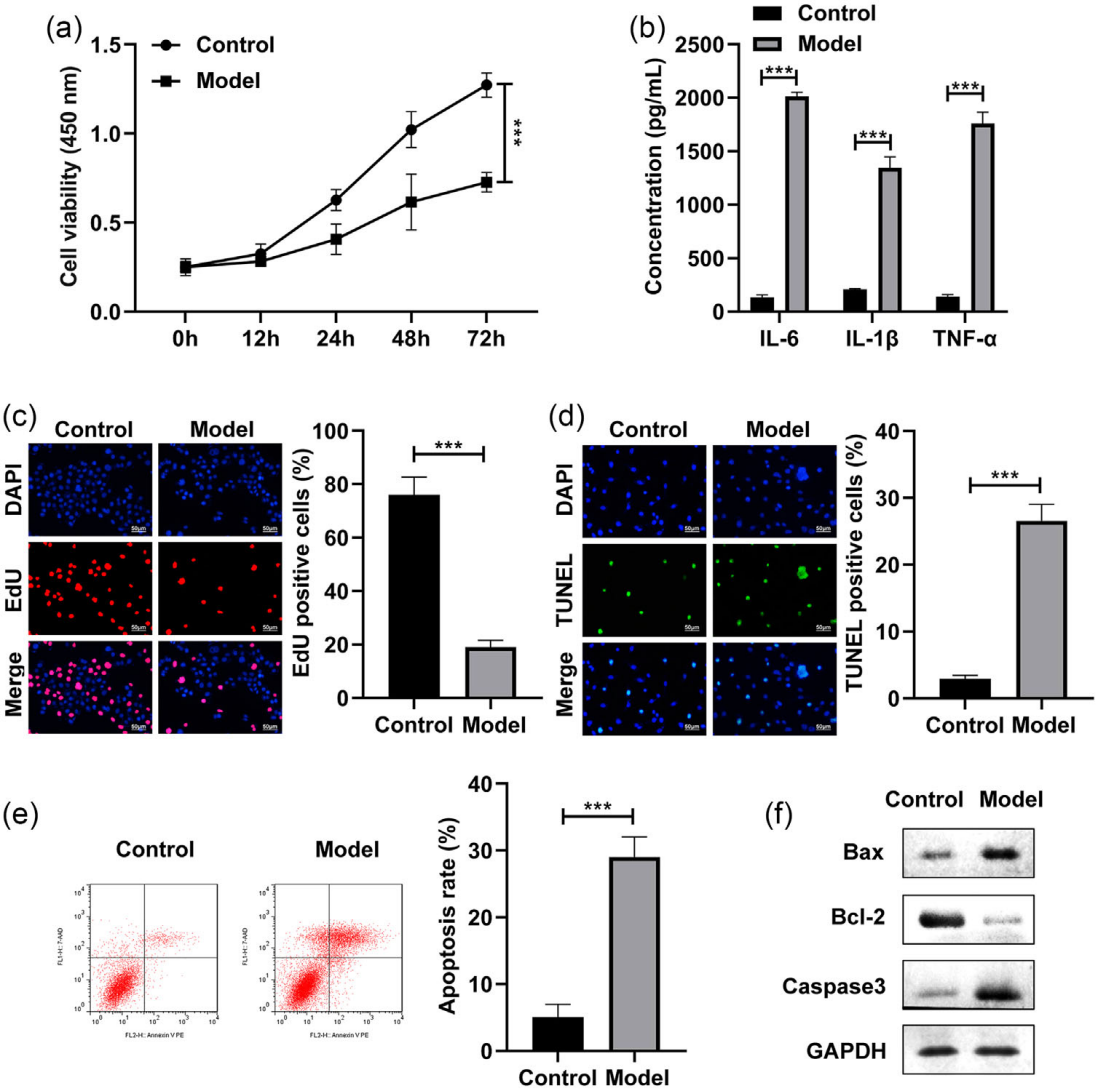
LPS stimulation inhibited the cell growth and induced the cell apoptosis of the SH‐SY5Y cells. The SH‐SY5Y cells were stimulated with LPS and exposed to 4% sevoflurane. (a) The cell viability was tested by MTT assay. (b) The proinflammatory factors were measured with ELISA kits. (c) Edu assay was performed to detect the cell proliferation. The cell apoptosis was analyzed by TUNEL staining (d) and flow cytometry (e). (f) The protein levels of Bax, Bcl‐2, and Caspase3 were detected by western blot. ***p < .001.

First, the SH‐SY5Y cells were stimulated by LPS and exposed to 4% sevoflurane to establish a POCD cell model. After LPS treatment, the cell viability of the SH‐SY5Y cells was dramatically inhibited (Figure 1a). Besides, the IL‐6, IL‐1β, and TNF‐α levels of the SH‐SY5Y cells were dramatically increased after LPS stimulation (Figure 1b). The EdU assay indicated that the cell proliferation was dramatically suppressed in the LPS‐stimulated SH‐SY5Y cells (Figure 1c). Furthermore, the cell apoptosis rate of the SH‐SY5Y cells were dramatically elevated after LPS stimulation (Figure 1d,e). Additionally, the Bax and Caaspase3 protein levels were significantly increased, and Bcl‐2 was decreased in the LPS‐stimulated SH‐SY5Y cells (Figure 1f). These results indicated the successful establishment of POCD model.

### 3.2 METTL3 was down‐regulated in the LPS‐stimulated SH‐SY5Y cells

**FIGURE 2:**
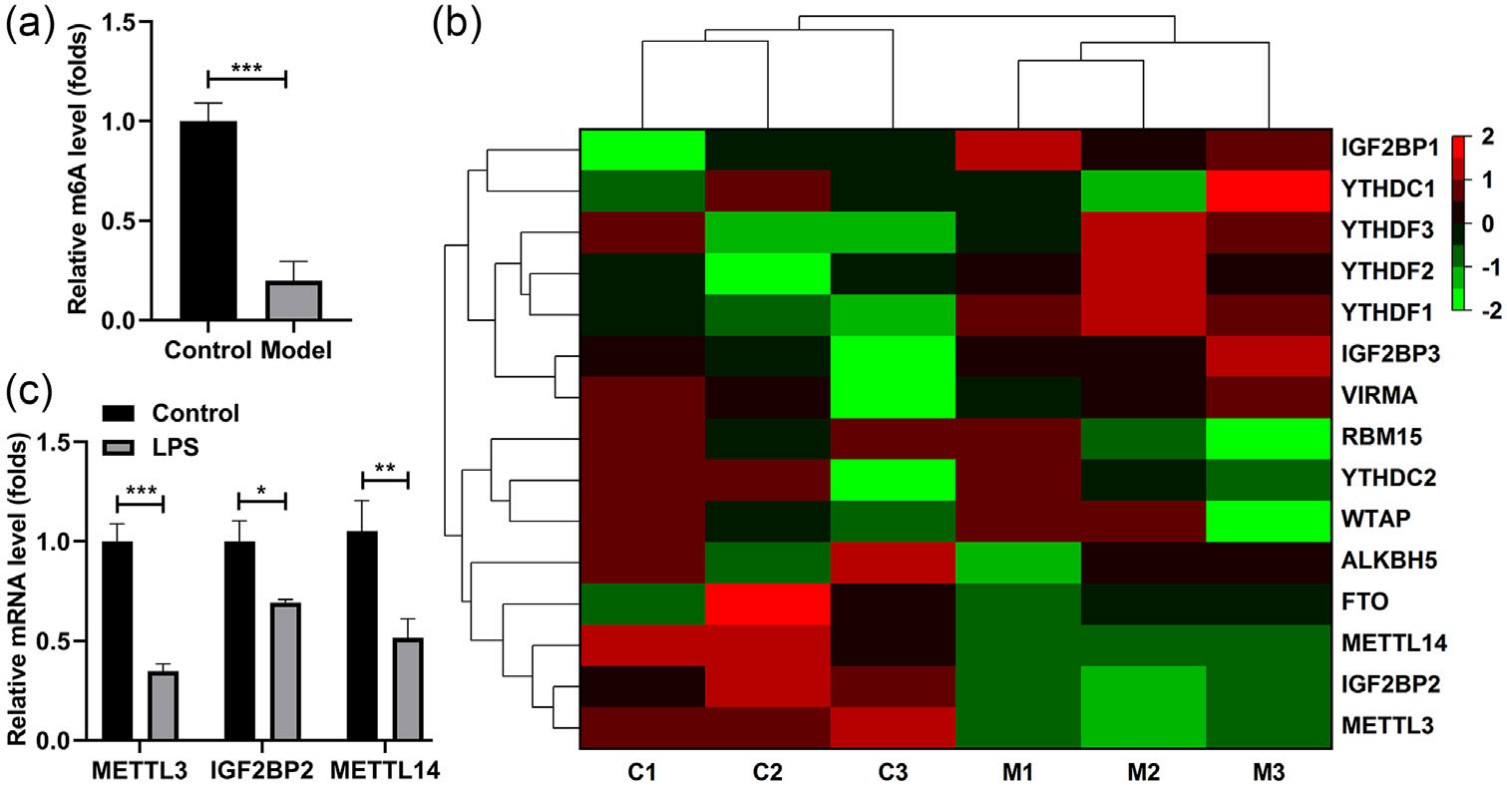
METTL3 was declined in the LPS‐stimulated SH‐SY5Y cells. The SH‐SY5Y cells were stimulated with LPS and exposed to 4% sevoflurane. (a) The m6A levels of cells. (b) The methylase levels were assessed by RT‐qPCR and expressed as heatmap. (c) The mRNA levels of METTL3, IGF2BP2, and METTL14 were quantified. *p < .05; ***p < .001.

Subsequently, we explored the m6A methylation levels in the LPS‐stimulated SH‐SY5Y cells. We found that the m6A levels were dramatically decreased after LPS stimulation (Figure 2a). Besides, we analyzed expressions of the m6A methylation‐related genes, which was expressed as the heatmap (Figure 2b). Among them, the top three downregulated genes were quantified, the expression of METTL3, IGF2BP2, and METTL14 in the LPS‐stimulated SH‐SY5Y cells was dramatically attenuated, and the METTL3 levels were the lowest (Figure 2c). Thereafter, we focused on the effect of METTL3 on the LPS‐stimulated SH‐SY5Y cells.

### 3.3 METTL3 overexpression alleviated the injury of SH‐SY5Y cells induced by LPS

**FIGURE 3:**
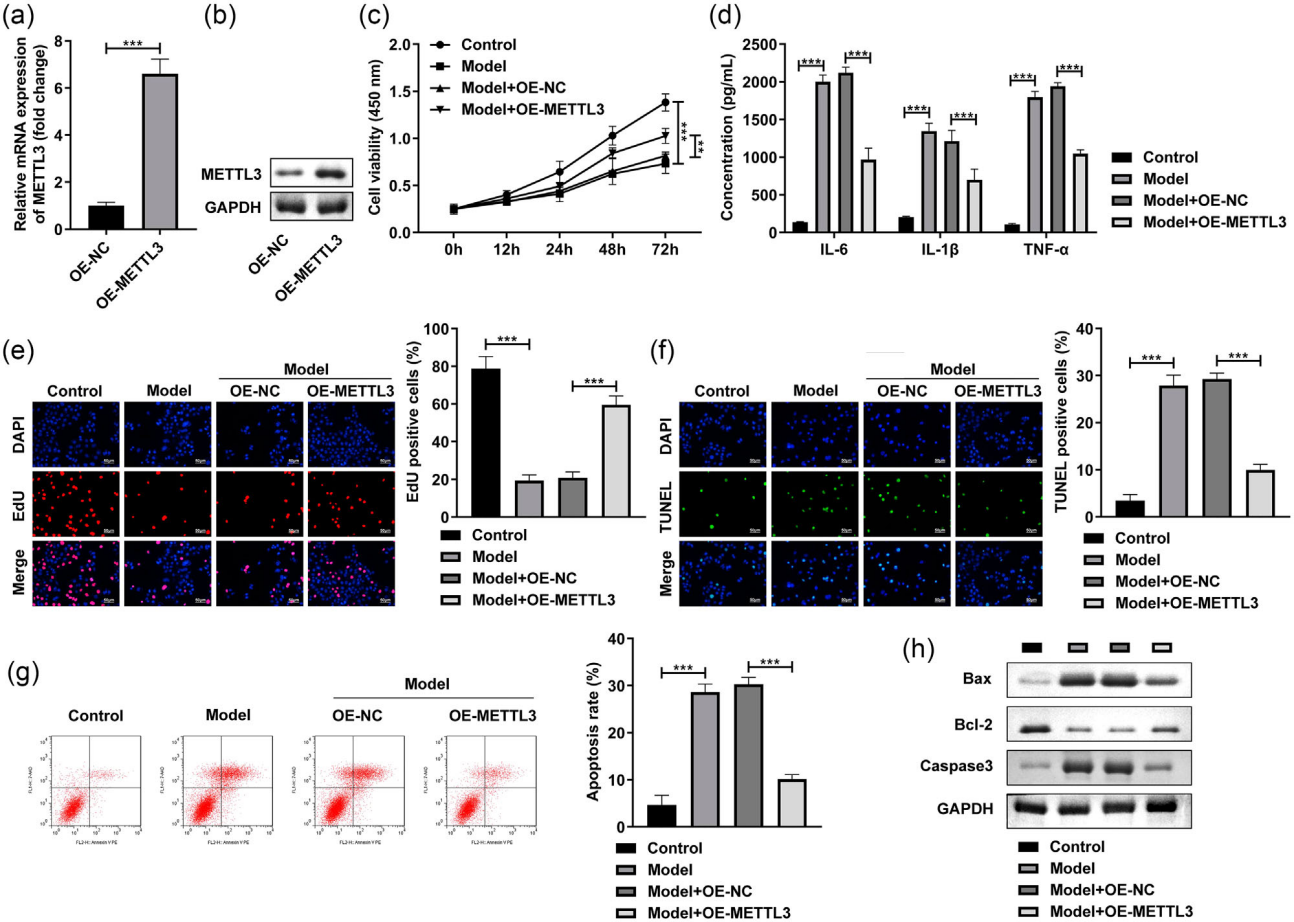
METTL3 overexpressed moderated the injury of SH‐SY5Y cells induced by LPS. The SH‐SY5Y cells were stimulated with LPS and exposed to 4% sevoflurane and transfected with OE‐METTL3. (a) The validation of transfection efficiency of OE‐METTL3 was performed by RT‐qPCR and western blot. (b) The cell viability was tested by MTT assay. (c) The proinflammatory factors were measured with ELISA kits. (d) Edu assay was performed to detect the cell proliferation. The cell apoptosis was analyzed by TUNEL staining (f) and flow cytometry (g). (h) The protein levels of Bax, Bcl‐2, and Caspase3 were detected by western blot. ***p < .001.

After OE‐METTL3 transfection, the METTL3 levels were dramatically enhanced at mRNA (Figure 3a) and protein levels (Figure 3a). Next, METTL3 overexpression prominently promoted the cell viability (Figure 3c) and reduced the IL‐6, IL‐1β, and TNF‐α levels of the LPS‐stimulated SH‐SY5Y cells (Figure 3d). Additionally, in the LPS‐stimulated SH‐SY5Y cells, the cell proliferation was prominently enhanced (Figure 3e), while apoptosis rate (Figure 3f,g) was reduced after METTL3 overexpression. Furthermore, MEETL3 overexpression significantly decreased the Bax and Caaspase3 protein levels, and increased the Bcl‐2 levels in the LPS‐stimulated SH‐SY5Y cells (Figure 3h). These findings implied that METTL3 might played critical role in the POCD progression.

### 3.4 METTL3 modulated the Sox2 levels in SH‐SY5Y cells

**FIGURE 4:**
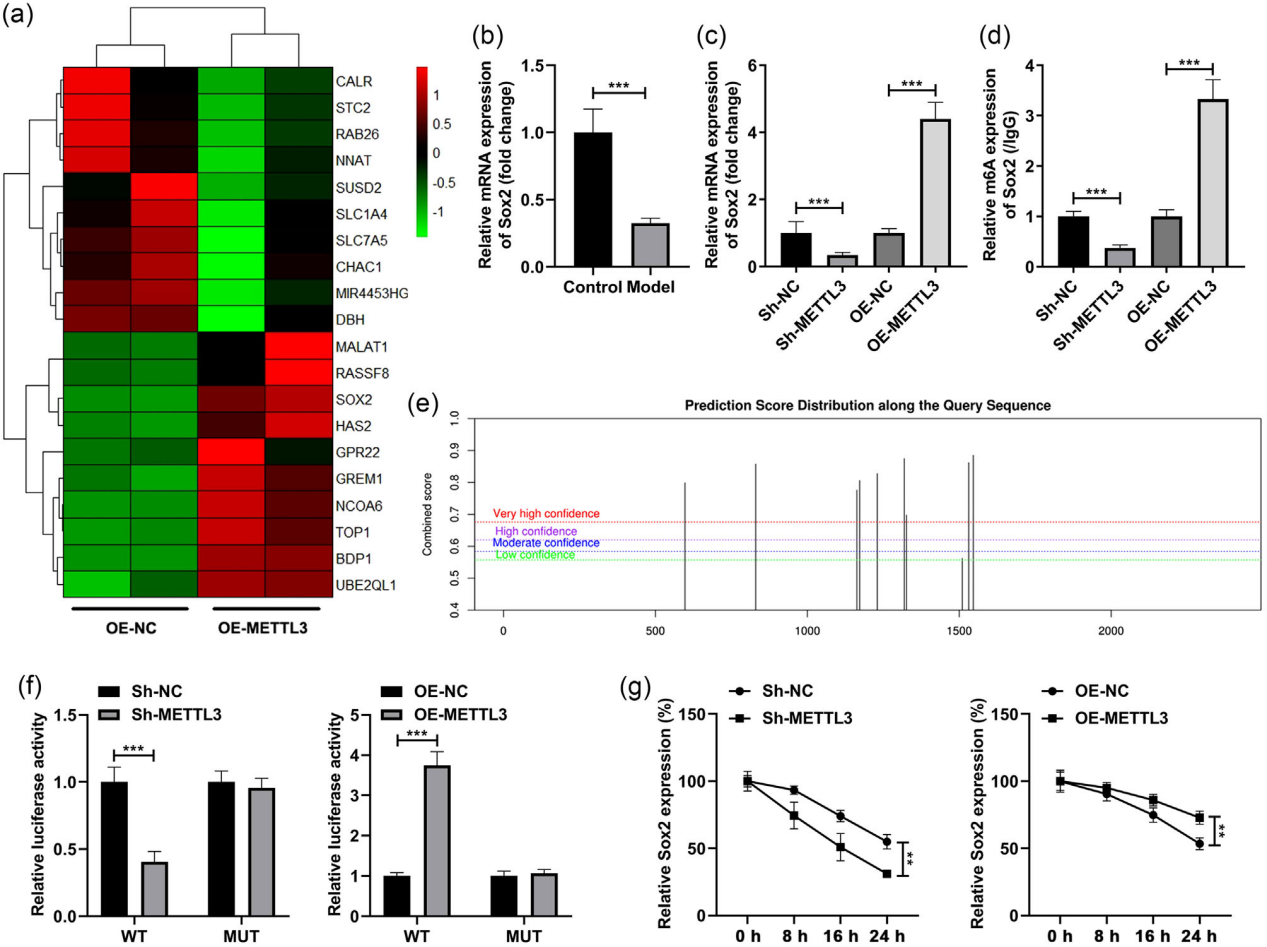
METTL3 modulated the Sox2 levels in SH‐SY5Y cells. (a) RNA sequencing was performed to analyze the differentially expressed genes in the SH‐SY5Y cells transfected with OE‐METTL3. The top 10 up‐ and downregulated genes were selected and expressed as heatmap. (b) The Sox2 levels in the LPS‐stimulated SH‐SY5Y cells were tested by RT‐qPCR. After OE‐METTL3 and si‐METTL3 transfection, the mRNA (c) and m6A (d) levels of Sox2 in the LPS‐stimulated SH‐SY5Y cells were tested by RT‐qPCR. (e) The m6(a) methylation sites of Sox2 were predicted using the SRAMP database. (f) The double luciferase report was performed to analyze the relationship between METTL3 and Sox2. (g) The stability of Sox2 was tested by RT‐qPCR. **p < .01; ***p < .001.

Through the RNA sequencing technology, we obtained the differentially expressed genes in the SH‐SY5Y cells transfected with OE‐METTL3. The top 10 up‐ and downregulated genes were selected and expressed as heatmap (Figure 4a). Among them, Sox2 was demonstrated to be closely relative to the learning and memory in POCD progression (Gui et al., 2021), which was selected for the next experiments. Besides, we found that Sox2 was prominently decreased in the LPS‐stimulated SH‐SY5Y cells (Figure 4b). Then, after METTL3 silencing, the mRNA and m6A levels of the Sox2 were prominently decreased, while METTL3 overexpression exhibited an opposite effect on Sox2 (Figure 4c,d). The bioinformatic analysis results showed that there are multiple methylation binding sites of Sox2 (Figure 4e). According to the prediction results, we synthetized the WT and MUT fragment (500–1600 bp) of Sox2 and inserted into luciferase reporter vector. The luciferase activity of WT‐Sox2 was prominently decreased after METTL3 silencing and enhanced after METTL3 overexpression, while no change in the luciferase activity was found in the cells transfected with MUT‐Sox2 (Figure 4f). Furthermore, the stability of Sox2 was prominently reduced after METTL3 silencing and enhanced after METTL3 overexpression (Figure 4g).

### 3.5 Sox2 silencing neutralized the functions of METTL3 in the LPS‐stimulated SH‐SY5Y cells

**FIGURE 5:**
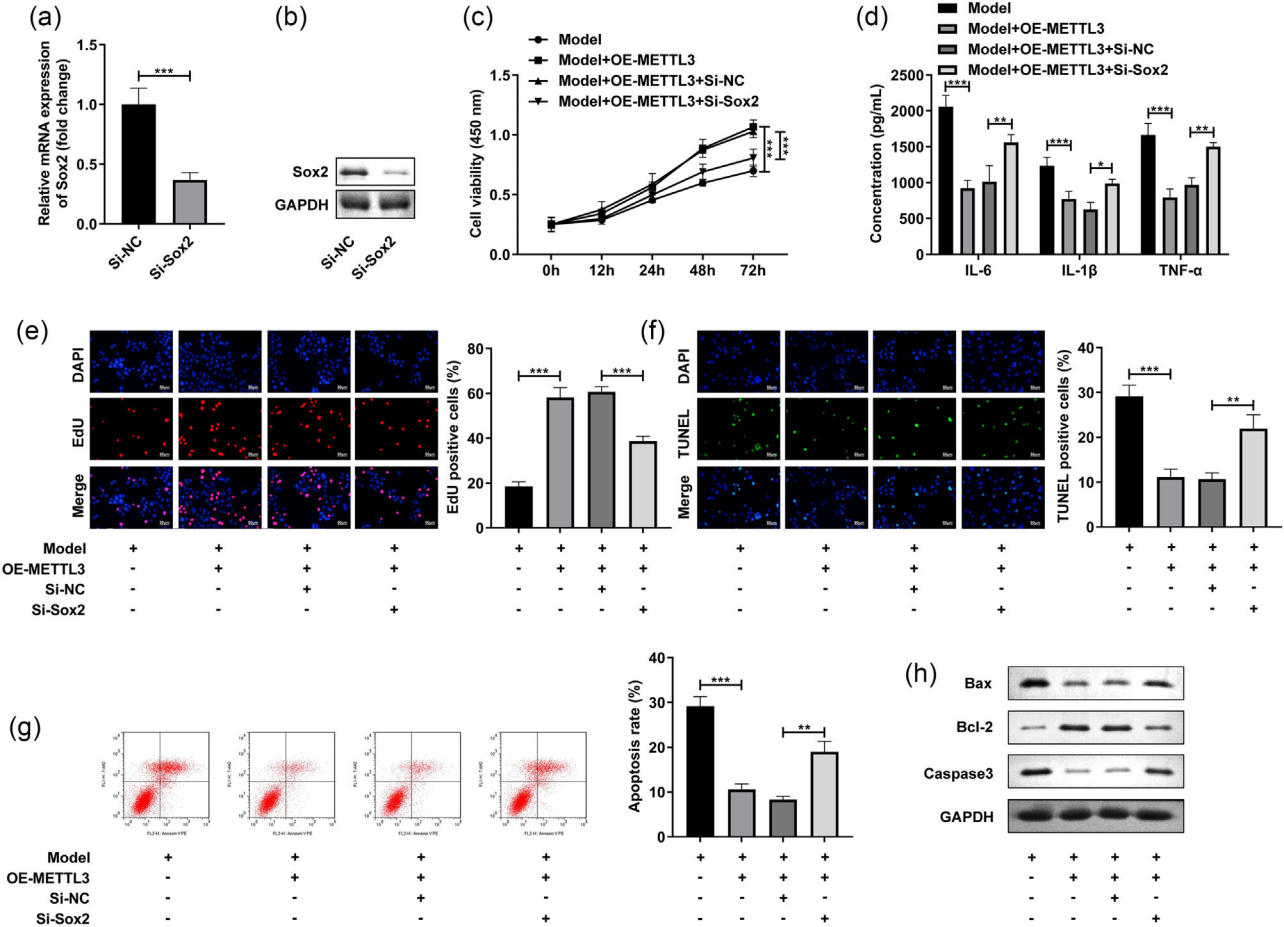
Sox2 overexpressed neutralized the functions of METTL3 in the LPS‐stimulated SH‐SY5Y cells. The SH‐SY5Y cells were stimulated with LPS and exposed to 4% sevoflurane and transfected with OE‐METTL3 and Si‐Sox2. (a, b) The validation of transfection efficiency of Si‐Sox2 was performed by RT‐qPCR and western blot. (c) The cell viability was tested by MTT assay. (d) The proinflammatory factors were measured with ELISA kits. (E) Edu assay was performed to detect the cell proliferation. The cell apoptosis was analyzed by TUNEL staining (f) and flow cytometry (g). (h) The protein levels of Bax, Bcl‐2, and Caspase3 were detected by western blot. *p < .05; **p < .01; ***p < .001.

Finally, we analyzed the role of Sox2 in the LPS‐stimulated SH‐SY5Y cells. The transfection of Si‐Sox2 prominently reduced the Sox2 levels in the SH‐SY5Y cells at mRNA and protein levels (Figure 5a). Besides, Sox2 silencing prominently reversed the increase of cell viability (Figure 5b) and elevated the decrease of IL‐6, IL‐1β, and TNF‐α levels caused by OE‐METTL3 in the LPS‐stimulated SH‐SY5Y cells (Figure 5c). Additionally, after Si‐Sox2 treatment, the cell proliferation (Figure 5d) was depleted and cell death (Figure 5e) and apoptosis rate (Figure 5f,g) were enhanced in the LPS‐stimulated and OE‐METTL3‐treated SH‐SY5Y cells. Besides, Sox2 knockdown significantly increased the Bax and Caaspase3 protein levels, and decreased the Bcl‐2 levels in the LPS‐stimulated and OE‐METTL3‐treated SH‐SY5Y cells (Figure 3h). These results implied that METTL3 participated in the POCD progression through regulating Sox2.

## 4 DISCUSSION

In the present study, we establish a POCD cell model through the LPS and sevoflurane stimulation. We found that METTL3‐mediated m6A methylation modification of Sox2 is involved in the regulation of POCD occurrence and development.

Recent researches have emphasized the critical role of RNA posttranscriptional modulation in brain functions (Zhao et al., 2017). It was reported that m6A participates in the regulation of cognitive function (Cheon et al., 2017; Deiner et al., 2021; Saletore et al., 2012). m6A methylation modification, as the most common RNA internal chemical modifier in eukaryotic cells, has been demonstrated to be involved in a variety of physiological and pathological functions, such as cell regeneration, osteogenic differentiation, spermatogenesis, fertility, and carcinogenesis (Ding et al., 2018; Jin et al., 2019; Yu et al., 2020; Zheng et al., 2013). At the same time, it also plays an important role in the regulation of nervous system functions, such as synaptic regeneration, synaptic function, and innate immunity of immune system (Weng et al., 2018). METTL3, as a classical methylase, has been proved to be involved in progressive neurological diseases. Zhang et al. (2022) found that METTL3‐mediated m6A participated in neuropathic pain progression, and demonstrated that METTL3 could be a molecular markers of neuropathic pain patients. Huang et al. (2020) confirmed aberrant METTL3 levels in the hippocampus of the Alzheimer's disease patients brain induced an epitranscriptomic mechanism, which resulted in the changes in related gene expression patterns in Alzheimer's disease. In this study, we found that METTL3 was lowly expressed in the LPS‐stimulated SH‐SY5Y cells, which resulted in the decrease of m6A levels of the whole cell. Besides, METTL3 overexpression alleviated the LPS‐induced cell apoptosis and inflammation in the SH‐SY5Y cells. These results preliminary supported that METTL3 plays critical roles in the POCD progression.

Sox2 is one of the markers of neural stem cells. As a transcription factor, Sox2 can regulate cell proliferation, migration, differentiation, and cell self‐renewal. SOX2 is first expressed in blastocysts, and as cells grow and develop, SOX2 is highly expressed in various tissues at later stages of development (Zappone et al., 2000). In the brain, SOX2 is widely present in neural precursor cells to promote the generation of the ventricular zone of the neural tube. SOX2 level is suppressed as cells migrate out of the ventricular zone (Mercurio et al., 2019). Recent researches demonstrated that structural or conditional ablation of Sox2 leads to developmental defects, depending on extent of Sox2 depletion (Hoefflin & Carter, 2014). Favaro et al. (2009) confirmed that decrease of Sox2 in nestin‐expressing cells results in an inability to maintain normal growth and development of the hippocampus, ultimately resulting in degradation of the hippocampal dentate gyrus. Additionally, previous researches have indicated that the decrease of Sox2 was observed in the injury of learning and memory (Mishra et al., 2012; Wang et al., 2018). These findings indicated the importance of Sox2 in the cerebral neurodevelopment. Similarly, we also demonstrated that Sox2 expression was prominently inhibited in the LPS‐stimulated SH‐SY5Y cells, which implied that Sox2 depletion was the key factor in POCD progression. It has been reported that Sox2 could be m6A modified by METTL3 in the breast cancer (Xie et al., 2021). Interestingly, we demonstrated that the m6A and mRNA levels of Sox2 were regulated by METTL3, and Sox2 silencing neutralized the OE‐METTL3 roles in the cell growth and inflammation in the LPS‐stimulated SH‐SY5Y cells.

In conclusion, this study demonstrated that METTL3 overexpression relieved the injury induced by LPS and sevoflurane treatment in the SH‐SY5Y cells through regulating the m6A and mRNA levels of Sox2. Our findings implied that METTL3‐mediated Sox2 may be a new choice as the target for the treatment of POCD in the future.

## AUTHOR CONTRIBUTIONS

All authors participated in the design, interpretation of the studies, analysis of the data, and review of the manuscript. Yilong Wang drafted the work and revised it critically for important intellectual content. Zeyong Yang was responsible for the acquisition, analysis, and interpretation of data for the work and made substantial contributions to the conception or design of the work.

## CONFLICT OF INTEREST STATEMENT

The authors declare no conflicts of interest.

### PEER REVIEW

The peer review history for this article is available at https://publons.com/publon/10.1002/brb3.2936.

## Data Availability

The datasets used and analyzed during the current study are available from the corresponding author on reasonable request.
